# Tolerance in Kidney Transplantation: What Is on the B Side?

**DOI:** 10.1155/2016/8491956

**Published:** 2016-11-10

**Authors:** Laura Carreras-Planella, Francesc E. Borràs, Marcella Franquesa

**Affiliations:** REMAR-IVECAT Group, Health Science Research Institute Germans Trias i Pujol, Can Ruti Campus, Badalona, Spain

## Abstract

Regulatory B cells (Breg) are in the spotlight for their role in immune homeostasis maintenance and tolerance achievement as in the last years the correlation with functional and increased Breg numbers in autoimmune diseases and transplantation has been extensively proven. Their study is, however, in its infancy with still little knowledge and consensus on their origin, phenotype, and mechanism of action. All this hampers the pursuit of an effective Breg induction method for therapeutic purposes. In this review we aim to summarize the studies on human Breg and their implication in kidney transplantation and to further discuss the issues surrounding therapeutic applications of this cell subset.

## 1. Introduction

Renal transplantation is the unique curative option for patients suffering from end-stage renal disease, but to date the evolution of each patient after transplantation cannot be predicted. In the past decades, acute graft rejection has decreased dramatically as a result of the introduction of immunosuppressive drugs. However, immunosuppressive drugs carry undesired and severe side effects such as infections, malignancies, and metabolic disorders [1] which may threaten patient's life. Yet, chronic rejection is still the main cause of long-term graft loss [2, 3]. The holy grail of organ transplantation is to maintain long-term graft function without immunosuppressive treatment, namely, operational tolerance (OT). However, OT is a rare event in kidney transplanted patients [4], as only about 0.03% of cases are estimated to be in such state [5]. Thus, despite the efforts made in the past, there is still a clear need to find new strategies to achieve long-term tolerance and to investigate the immunological mechanisms that may be implicated in the process of OT.

Among the actors implicated in the mechanisms of the immune response, B and T lymphocytes are the main characters that lead to graft rejection. In this play, B lymphocytes have a dual key role since they present antigens of the donor to T cells in addition to secreting antibodies that can lead to acute rejection or, later in time, chronic rejection [6]. Nevertheless, a sparse B cell subset has been attributed immune regulatory functions which conveys that not all B cells play on the rejection side. Although it was first described in 1974 [7] it was not until 2000 that this population was named regulatory B cells (Breg) [8]. In the last decade, the regulatory role played by Breg has been highlighted by many authors in autoimmune diseases such as systemic lupus erythematosus (SLE) [9], rheumatoid arthritis [10], and pathologies that promote antineutrophil cytoplasmic antibodies [11] and also in allograft tolerance in organ transplantation [12, 13]. The current general consensus is that Breg develop their function mainly via the secretion of IL-10 [14, 15]. However, a complete phenotype signature, development pathway, or the immunoregulatory properties of Breg have not been fully discovered in mice nor in humans, thus granting future research on this cell type.

In this review, our aim is to gather the current knowledge about regulatory B cells and their role in kidney transplantation tolerance in humans and to discuss their potential application as cellular therapeutic agent.

## 2. Regulatory B Cells: Phenotype and Function

One of the darkest spots of Breg is their phenotype, since for years researchers in the field have tried through multiple approaches to find unique characteristic markers to define them. However, there is still no consensus on it. There is less discussion about their mechanism of action, which is principally accepted to be IL-10, but the lack of knowledge on what triggers its secretion and the fact that other regulatory mechanisms have also been proposed leave this issue, to date, unresolved.

### 2.1. Does a “Unique” Breg Phenotype Exist?

As previously occurred in the studies on regulatory T cells, many researchers have prompted to identify a unique set of markers, transcription factors, or mechanism of action that exclusively identify Breg in all contexts. In this sense, genetic and surface expression studies have been conducted with partial success to unravel a unique Breg signature [16, 17]. Unfortunately, to date such unequivocal markers have not been found yet. Also, some hypothesis have been formulated on Breg development pathways from a common precursor [18, 19], but the results so far are not conclusive. Thus, most authors rely on the capacity to produce interleukin- (IL-) 10 and on the two main phenotypical signatures used to define Breg: (1) transitional B cell phenotype CD19^+^CD24^hi^CD38^hi^ and (2) CD19^+^CD5^+^CD1d^hi^ (used in both human and mice) [20, 21]. Nevertheless, we still face a lack of specific Breg markers, and different phenotypes for IL-10-producing B cells with regulatory capacity have been proposed through the years. In 2008, Yanaba and colleagues identified an IL-10-producing regulatory B cell subset in mice expressing CD1d^hi^CD5^+^ which they referred to as B10 cells [21]. A few years later, the same group characterized a similar IL-10-producing B cell subset in humans. Human B10 cells' regulatory potential was shown by their capacity to inhibit tumor necrosis factor- (TNF-) *α* production by CD4^+^ T helper cells and monocytes. In peripheral blood, B10 cells were found exclusively among CD24^hi^CD27^+^ cells, whereas in spleen no difference was observed between IL-10-producing and nonproducing B cells regarding their surface markers [15]. When testing the immunomodulatory capacity of the same subset from patients with allergic asthma* in vitro*, these cells were less capable of secreting IL-10 and inducing the secretion of IL-10 from CD4^+^ T compared to the same cell population from healthy subjects, suggesting that this population could hold immunomodulatory capability [22]. Nevertheless, Matsumoto et al. found that CD27^int^CD38^+^ immunoglobulin- (Ig-) secreting plasmablasts that arise from naïve and immature B cells from human blood are the major IL-10-producing B cells after* in vitro* stimulation [23]. Yet, the transitional B cell subset CD24^hi^CD38^hi^ also seems to have regulatory capacity, since after CD40 stimulation they could suppress the differentiation of naïve T cells into T helper 1 (Th1) and Th17 and lead CD4^+^CD25^−^ T cells conversion into regulatory T cells (Treg), partially via IL-10 [9, 24].

Due to the disparity of the results showing that different B cell subsets can express immunomodulatory properties, a current emerging view is that Breg are not a specific B cell subset but rather a circumstantial B cell phenotype. In this scenario, B cells could acquire a regulatory role when appropriate signals are generated in the environment, as has been already suggested by some authors [25–27]. It would seem reasonable to think that, depending on the type of activated immune cells and cytokines released to the environment, some B cells could shape their response towards the appropriate way to modulate the response of other immune cells. The adaptability of the Breg response could explain the different outcomes depending on the disease studied* in vivo* or the stimulation provided* in vitro*. Therefore, maybe the quest for a “unique” Breg marker must be reoriented to find the right stimulation for B cells to become stable regulators of the immune response in a given scenario.

### 2.2. IL-10 Secretion as Breg Mechanism of Action

As mentioned above, IL-10 production is perhaps the principal hallmark to define regulatory B cells, describing their immunomodulatory potential and explaining their mechanism of action. IL-10 is a regulatory cytokine secreted by almost all innate and adaptive immune cells that plays an essential role in maintaining immune homoeostasis [28]. It binds as a homodimer to its receptor which is a tetramer formed of two *α* (IL-10R1) and two *β* (IL-10R2) chains. IL-10R1 binds to the cytokine while IL-10R2 is responsible for the downstream signaling activation through Jak1 and signal transducer and activator of transcription 3 (STAT3). IL-10 is the only ligand for IL-10R1 which in turn is the unique receptor of IL-10, while IL-10R2 is shared by several cytokines such as IL-20, IL-22, IL-24, IL-26, IL-28, and IL-29 [29]. Although little is known about the molecular pathways involved in IL-10 secretion in humans, in mice it is mediated by store-operated Ca^2+^ influx from the endoplasmic reticulum, which is further regulated by the calcium sensors stromal interaction molecule (STIM) 1 and STIM2 [30].

**Figure 1:**
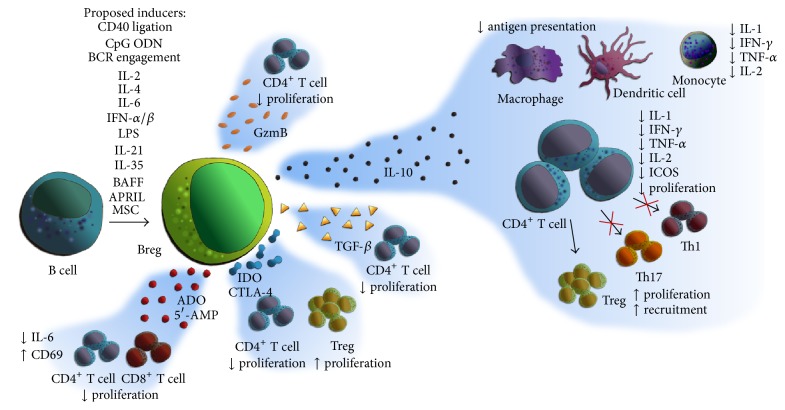
Graphical summary of the main Breg induction methods, mode of action, and immunosuppressive functions that have been proposed in the literature. ODN: oligodeoxynucleotides; IL: interleukin; BCR: B cell antigen receptor; IFN: interferon; LPS: lipopolysaccharide; BAFF: B cell activating factor; APRIL: a proliferation-inducing ligand; MSC: Mesenchymal Stem Cells; GzmB: granzyme B; TGF: transforming growth factor; ADO: adenosine; AMP: adenosine monophosphate; IDO: indoleamine 2,3-dioxygenase; CTLA-4: Cytotoxic T-Lymphocyte Antigen 4; ICOS: inducible T cell costimulator.

Among other biological functions, IL-10 promotes the downregulation of antigen presentation by macrophages and dendritic cells and suppresses the production of proinflammatory cytokines such as IL-1, interferon (IFN)-*γ*, and TNF-*α* by CD4^+^ T cells, monocytes, and macrophages [29, 31] (Figure 1).

Besides the functional relevance of IL-10 expression, IL-10-producing CD19^+^CD24^hi^CD38^hi^ B cells have also been shown to promote the expansion of IL-10-producing FoxP3^+^ Treg and to play a role in inducing their recruitment to the site of inflammation [32]. In addition, human IL-10-producing B cells may block the CD28^−^ inducible T cell costimulator (ICOS) costimulatory pathway, thus blocking T cell activation via phosphorylation of Src homology region 2 domain-containing phosphatase-1 (SHP1), a downstream molecule of the IL-10 receptor intracellular pathway [33]. Moreover, they regulate innate immune responses by reducing TNF-*α* production by monocytes [15].

The key role of IL-10 released by B cells has been also proven in multiple sclerosis (MS) patients, who have B cells with impaired IL-10 production under CD40 stimulation [34]. Similarly, in SLE patients, B cells fail to produce IL-10 in response to CD40 but not to CpG oligodeoxynucleotides (CpG) [9]. These results indicate an impaired T cell-dependent Breg induction in both autoimmune diseases.

### 2.3. IL-10 Independent Regulatory B Cells

Not only IL-10 but also other regulatory mechanisms like IL-35 [35], granzyme B (GzmB) [36], transforming growth factor- (TGF-) *β*, and indoleamine 2,3-dioxygenase (IDO) [37] have been suggested as important molecules in Breg tolerogenic function. Figure 1 depicts some of the different Breg inducers, mechanisms of action, and functions described in several studies. In one of them, when CD4^+^CD25^−^ T cells were cocultured with CD40-CpG-stimulated B cells from either healthy controls (HC), immunosuppressive-dependent stable graft function (SI) patients, or OT patients, the proliferation of T cells was inhibited. When IL-10, TGF-*β*, or GzmB were blocked separately, only the anti-GzmB antibody hindered the inhibitory effect on T cell proliferation [12]. However, in a similar experimental set-up where B cells were stimulated with CpG alone, the blockade of TGF-*β* and/or IDO activity led to decreased antiproliferative function of Breg in coculture with T cells [37], suggesting different immunosuppression mechanisms depending on the stimulation.

Somehow these papers entail an IL-10 alternative immunosuppressive mechanism of action rather than a characterizing feature of Breg. This might hold true for determined Breg subsets and strengthens the idea of diverse Breg phenotypes depending on the environment.

## 3. Potential Role of Regulatory B Cells in Transplantation

Due to their central role as effector cells in the immune response, particularly in acute organ rejection, T lymphocytes have been one of the main targets of immunosuppressive treatments. B lymphocytes also participate in acute rejection by infiltrating allografts and presenting alloantigens to T lymphocytes, promoting the production of IFN-*γ*, IL-4, and IL-6 among others cytokines. These cells are also capable of differentiating into plasma cells, switching from antigen presenting cells to antibody secretory cells that may target MHC class I and II molecules of the graft. This process occurs latter in time and usually leads to chronic rejection [38]. To hamper this process, an anti-CD20 B cell depleting monoclonal antibody, rituximab, has been introduced as immunosuppressive treatment for transplanted patients. Despite the fact that the use of this drug has increased patients' survival, it fails to induce chronic unresponsiveness to the graft [39, 40]. One of the possible reasons underneath may be that plasmablasts and plasma cells, two key players in chronic rejection, do not express CD20 on their cell surface. An additional explanation may be that Breg are also depleted by the treatment, thus hampering their tolerogenic function. In this sense, some studies have shown that preserving the B cell compartment favors OT in renal transplantation [41].

### 3.1. Breg as a Tolerance and Good Prognostic Biomarker in Kidney Transplantation

Seminal papers coled by US and UK consortia (IOT, RISET, and ITN) [42, 43] showed a similar transitional-Breg-related gene signature corresponding to immunosuppressant-free spontaneous OT kidney transplant patients. Using microarray analysis and real time PCR, they identified a B cell specific gene signature and different B cell subpopulations distribution in OT patients compared to SI patients after transplantation. The signature proposed by Newell et al., relating OT patients to HC but not to their IS counterparts, includes 30 genes, most of them are encoding for the *κ*/*λ* light chains of Ig. In the cross-validation experiments, three of these genes were found to be the most predictive: IGkV1D-13, IGLL1, and IGkV4-1. On the other hand, flow cytometry analyses revealed an increased number of total and naïve B cells in OT with respect to SI patients. Transitional B cells (defined by the group as CD19^+^CD24^+^CD38^+^IgD^+^) were also found to be increased in tolerant patients and that was consistent in both ITN and IOT cohorts. Since then, several other groups have showed similar traits in their OT or SI patients [13, 44]. Chesneau et al. reported that tolerant patients showed a higher frequency of transitional (defined as CD20^+^CD24^hi^CD38^hi^) and naïve (defined as CD20^+^CD24^lo^CD38^lo^) B cells and a higher production of IL-10 compared to SI patients [45]. In line with this observation, patients with chronic antibody mediated rejection after renal transplantation were found to have less percentage and absolute numbers of transitional B cells (defined as CD19^+^CD24^hi^CD38^hi^) when compared to the group of SI patients [46].

A recently published update of the ITN study revealed a maintained gene signature among OT patients but surprisingly the gene set also increased over time in those SI patients. Flow cytometric analysis of the B cell population shows a persistent increase in total, naïve, and transitional B cell population in OT compared to SI patients [47].

Furthermore, additional studies have compared transitional/Breg frequencies in OT, SI, HC, and also chronic rejection patients. Interestingly, the last group shows low levels of transitional B cells comparable to the ones of SI patients [13].

Drawing on the correlation between kidney transplant tolerance and regulatory B cells, the prognostic value of pretransplantation transitional/regulatory B cells and transplantation outcome has been approached [48, 49]. In a prospective study, Shabir and colleagues show that only higher transitional B cell frequencies before transplantation, but not regulatory T cells, total B cells, or memory B cells, correlate with lower incidence of biopsy proven acute rejection [48]. Moreover, patients lacking transitional B cells three months after transplantation are at higher risk of suffering from both T cell and antibody mediated rejection [49].

Altogether these studies suggest a marked role of the transitional B cell compartment in graft acceptance and tolerance achievement, which implies that transitional B cells and Breg are at least partially overlapping populations. It still remains unclear whether the tolerogenic effect is only created by the “natural” Breg present in the recipient or whether they can be induced in any patient to generate a tolerance status.

### 3.2. Current Immunosuppression Regime and Breg Induction

Since Breg and transitional B cells have been acknowledged as a key cell type in the induction and maintenance of tolerance, several groups have studied the effect of different treatments on these B cell compartments in the human setting.

The study of the B cell subsets profile in patients under different immunosuppressive regimes has been approached by some groups. The results reported so far have demonstrated that neither mTor nor Calcineurin inhibitors (CNI) induce transitional nor regulatory B cells [50–52]. Although mTor inhibitors have shown Treg inducing capacity, this effect seems to be Breg independent. Further, the* in vitro* study of the effect of CNI revealed that it inhibits IL-10 expression of B cells [50].

Other immunosuppressive agents, such as the B cell depleting antibodies alemtuzumab (anti-CD52) and rituximab (anti-CD20), have also been tested in transplant patients for their capacity to induce Breg. Alemtuzumab treated patients show a transient increase in transitional B cells along with a sustained increase in naïve B cells [53]. Conversely, rituximab has produced far more controversial results. While a single prophylactic dose seemed to protect from developing acute cellular rejection [54] and even induce a B cell repopulation based on transitional B cells [55], a clinical trial using two doses of the same compound on days 0 and 7 after transplantation had highly deleterious effects, causing excessive rates of acute cell rejection which forced the premature termination of the trial [56]. These studies may suggest that there is a window of time- and dose-dependent effect of B cell depletion to induce regulatory or effector B cell subsets in patients under these treatments.

Finally, next generation blockers of the B cell function which are being approached in autoimmune diseases, such as belimumab (B cell activating factor (BAFF) blocker) or atacicept (transmembrane activator and CALM interactor (TACI) blocker, affecting both BAFF and a proliferation-inducing ligand (APRIL)), will undoubtedly also have an effect on the B cell profile of patients, but to date there is no information on their effect on the Breg population.

Beyond conventional immunosuppressive treatments, other nonconventional approaches have also proven Breg induction potential. Mesenchymal Stem Cells (MSC) therapy is one of the leading nonpharmacological therapies in transplantation. Several clinical trials have approached their tolerogenic potential and a few brought their attention to Breg induction. Patients with refractory chronic graft versus host disease (cGvHD) present lower frequencies of total B cells and CD5^+^IL-10^+^ B. However, after three months of MSC treatment patients showed improvement of their symptoms correlating with increased CD5^+^IL-10^+^ B cells. Of note, the plasmatic levels of IL-10 were also higher after the treatment in these patients [57]. In another phase II multicenter clinical trial, lymphocyte subsets were analyzed in patients infused several times with umbilical cord-derived MSC to treat cGvHD. Although there were no differences between the control and the treated groups regarding B cell numbers, the number of particular CD27^+^ B cells was higher in the treated group after some months of MSC infusions, and the clinical symptoms improved [58].

An additional way to induce IL-10 and functional Breg may rely on helminths infections [59]. Individuals infected with* Schistosoma haematobium* have higher percentage of IL-10 producing B cells that are able to induce Treg and IL-10 production by T cells in coculture. Moreover, helminthic infection of MS patients has shown therapeutic potential since those patients that were infected presented less clinical symptoms compared to noninfected MS subjects [60]. The authors determined that B cells from helminths-infected MS patients produced more IL-10 than noninfected MS patients, and that these IL-10 producing cells had a phenotype similar to naïve B2 cells.

Altogether these studies show the potential of several compounds and therapeutic approaches to induce Breg. However, the significance of the increase of this particular B cell subset and their specific role in the progression of the disease or the therapeutic effect still need to be fully determined. Hence, a proper knowledge on Breg is mandatory to monitor the efficacy of the treatment as well as the tolerogenic status of the patient.

## 4. Breg as Cell Therapy

In view of the potential of Breg, many efforts have been made trying to find out how to effectively induce Breg* in vivo* and to deepen into the mechanisms of action underlying Breg induction.  Table 1 summarizes the principal described methods in human samples. This vast knowledge is of paramount importance to get more insight into the potential mechanisms and therapeutic targets to induce Breg* in vivo*, strategies for* ex vivo* induction for forthcoming cell therapy-based approaches, and purification of Breg for their further study and characterization.

### 4.1. Purification of B Cells for Breg Induction

The cell source to purify B cells and to produce Breg* in vitro* differs from one lab to the other. While most groups use peripheral blood mononuclear cells as the main source of B cells due to the easy accessibility of blood, other sources such as lymph nodes (i.e., per indication from removed tonsils) or spleen (i.e., discarded organ from cadaveric organ donor) may also be important to get even larger numbers of B cells. Although the levels of expression of some surface markers could vary between B cells from different compartments [67], little is known about how this can affect the induction of Breg in* in vitro* experiments.

The purification of the B cells may also be approached using different methodologies. Most laboratories use positive selection with CD19 antibodies. CD19 is expressed from the early pro-B cell stage to the B cell lymphoblast stage, but the expression is downregulated upon B cell maturation to plasma cells. Aiming at minimizing B cell activation induced by CD19 ligation, many other groups use CD19 negative selection to purify B cells. CD43 is expressed on activated B cells, plasma cells, CD5^+^ B-1a cells, and non-B cells, thus resulting in a good marker to isolate untouched resting mature B cells. Alternatively, CD22 is expressed on the surface of mature B cells in peripheral blood, but not on plasma cells or early stages of B cell differentiation [68], resulting in the isolation of untouched CD19^+^ B cells.

### 4.2. *In Vitro* Expansion of Breg

As the only current defining characteristic of regulatory B cells is their capacity to secrete IL-10, induction of Breg from B cells is usually measured based on the proportion of IL-10-producing B cells. Although the intracellular pathways are not well known yet, they seem to be inducible in different ways. Ligation of CD40, B cell antigen receptor (BCR), and/or toll-like receptors (TLR) together with IL-2 or IL-4 are the most used stimulating factors. However, a consensus regime to induce IL-10 producing Breg is still to be defined. Decreased expression of TLR9 due to polymorphisms in the tlr9 gene can increase predisposition to SLE in humans [69], which suggests that this is a key factor in Breg induction. TLR9 ligation to induce Breg can be achieved basically with CpG type B (generally 2006). Lipopolysaccharide (LPS), which ligates to TLR4, is more frequently used in mice since human B cells express very low levels of this receptor in physiological conditions. However, stimulation with anti-IgM, CD40L, and IL-4 can increase TLR4 expression by human B cells [70]. This may explain why Iwata et al. [15] found that both LPS and CpG induced IL-10^+^ B cells and CD40 ligation enhanced this effect.

Also, APRIL has been demonstrated to promote the generation of IL-10 producing B cells via STAT3 induction [66]. Compared to naïve B cells from peripheral blood, naïve B cells from cord blood seem to have a higher capacity to produce IL-10 after stimulation, which could be related to a higher level of pSTAT3 after CD40 stimulation [65]. This observation reinforces the importance of the source of B cells for* ex vivo* expansion.

Another strategy to induce Breg* ex vivo* is the use of MSC. Our group has recently demonstrated that MSC support B cell survival and have a direct effect on their differentiation. When B cells derived from tonsils were stimulated with BCR plus CD40 ligation in the presence of IL-2, plasmablasts were induced. But when B cells in the same setting were cocultured with MSC derived from adipose tissue, plasmablast formation was abrogated and Breg (CD19^+^CD24^hi^CD38^hi^IL-10-producing B cells) were induced [63]. Similar results were obtained using B cells from blood, as when they were cultured together, MSC promoted the survival and proliferation of B cells and increased the CD5^+^ B cell subset, which has also been described to have immunoregulatory capacity. Even though the mechanisms underlying these effects are unknown, in the same study they showed that inhibition of the IDO pathway partially reduced the effect of MSC on B cells, while blockade of COX-2/Prostaglandin-E2 pathway, IL-6, or IL-10 did not have any effect [57].

### 4.3. Breg Based Cell Therapy

Based on the studies summarized in this review, and also in other studies that have not been mentioned due to space limitation, it is clear that Breg may be envisaged as an additional approach for promoting tolerance in several pathologic situations.

Cell therapy is not a new concept anymore and even in the solid organ transplantation field protocols and clinical trials are being set up to promote tolerance in the absence or in a minimized immunosuppressive regime. MSC therapy has taken the lead in this area with several trials done and published in kidney, liver, and bone marrow transplantation. In parallel, regulatory immune cell types such as regulatory T cells, tolerogenic DCs, or regulatory macrophages are the main immune cell types being studied and used for cell therapy in human organ transplantation. The ONE study, a cooperative project that aims at developing immunoregulatory cell therapies for organ transplanted patients [71], is the paradigm since they compile and share the knowledge among the research groups devoted to that field. However, to this moment, there are no trials on the use of Breg as a cell therapy. The incomplete knowledge on Breg induction, stability, and functional potential and the lack of a consensus Breg signature are just some of the hurdles to be bypassed to generate a safe and efficient cell product. We might be dealing with different subsets of Breg depending on the induction cocktail and system used that might present different stability and functionality.

Since most of the induction systems used at this moment promote activation of B cells, Fillatreau and colleagues propose a method to induce IL-10 expression on resting B cells to generate tolerogenic B cells which are poor immunogenic and present a lower potential risk of switching into effector B cell [72]. Another matter of concern is the antigen specificity of Breg. In contrast to dendritic cells, B cells cannot phagocyte an antigen to present it on their surfaces but instead it needs to be recognized by specific BCR, internalized, and presented in MHC-II [73]. It is unknown whether this antigen is inducing specific tolerance, but if this was the case, it would be necessary to find out how to generate antigen-specific Breg. New technical advances in nanosciences might bring new opportunities into that area.

The effect that donor or recipient-derived Breg could have in modulating the immune reaction remains unknown if we envision a therapy in the field of organ transplantation or the effect of autologous or allogeneic Breg in autoimmune diseases. Identifying the mechanism of action by which one and not the other could induce allograft tolerance can shed light on the role of direct and indirect pathway of antigen presentation and tolerance induction. Moreover, the age of the patient is a relevant factor in the capacity of regulatory B cells to produce IL-10 since it is impaired in CD38^hi^CD24^hi^ B cells from old individuals independently of the stimulating factor used (CD40L, phorbol 12-myristate 13-acetate (PMA)/ionomycin, or CpG) [74]. This might be a major problem if we think of autologous cell treatment. There are other mechanistic issues that would have to be addressed such as the time needed to produce enough Breg, infusion timing and dosage, route of administration, and GMP compliance.

In addition to all the above mentioned issues, we are facing an added difficulty in the development of such a therapeutic strategy: the model. In mice models, multiple studies show that Breg can induce Treg and are capable of transferring tolerance in allogeneic cardiac allograft [75], islet allograft [76], and arthritis [77] models. These studies point out the central role of IL-10 in the modulation of the immune response. Also, another study with islet allograft full MCH-mismatched model suggested that Treg induction by Breg could be mediated through TGF-*β* [78]. In a rat cardiac allograft model, transferred Breg from tolerant animals migrated to the graft where they maintained their regulatory capacity [79]. Back to mice, T cell Ig mucin protein-1 (TIM-1) also seems to be a key molecule for Breg since TIM-1^+^ B cell subset is highly enriched for IL-10 producing cells, and the secretion of IL-10 increases substantially after TIM-1 ligation [80]. Nevertheless, it is proven that murine and human Breg are essentially different and that their* ex vivo* induction involves different mechanisms and molecular pathways. In this scenario, we will have to rely on human cell culture approaches and humanized mouse models in order to develop a therapeutic strategy.

Besides the generation of therapeutic protocols to induce Breg* in vivo* and taking into account the achievements in* in vitro* expansion of Breg, one can envision in the near future a cell therapy approach using Breg to promote tolerance.

## 5. Conclusions

Regulatory B cells are one of the newest members of the regulatory immune cells family. Many researchers in the field of transplantation and autoimmune diseases have turned their attention to this cell type for their implication in maintaining homeostasis and achieving a tolerant state.

While patients suffering from autoimmune diseases such as SLE or MS have shown nonfunctional Breg populations [9, 81] in the transplantation field, higher pretransplantation Breg numbers have been associated with lower antibody mediated rejection in kidney transplant recipients [48]. Further, in kidney transplantation, Breg have become highly interesting due to their association with tolerance [12, 42, 43]. However it still remains elusive whether the increase in Breg is cause or consequence of the tolerance status [82].

Despite the increasing number of papers published about Breg, one of the main hurdles in their study is the absence of a Breg signature, and the fact that murine Breg are substantially different to the human ones is hindering this endeavor. Until now the human Breg signature has been mainly resolved by the use of transitional B cell phenotype and/or the ability to secrete IL-10 but other extracellular markers and released cytokines have been associated with this cell type. The variability of the Breg signature might be due to different Breg phenotypes depending on the disease, the activation milieu, or the cell origin. Unraveling a proper set of markers that identify this regulatory subset will help in the monitoring of patients and will bring new light into their relation with the immune homeostasis.

In any case, functional Breg seem to be directly involved in graft tolerance. The generation of a Breg* in vitro* might be the key point to regain the lost tolerance status opening new doors to the development of innovative therapies.

## Figures and Tables

**Table 1:** Summary of the recently used Breg induction methods in human *in vitro*.

Isolation^*∗*^	Stimulation	Characterization of induced Breg	Immunosuppressive effect (mechanism of action)	Reference
CD19 positive selection	CpG (10 ug/mL) + CD40L (1 ug/mL) for 24 hours	CD24^hi^CD27^+^IL-10^+^	Inhibition of TNF-*α* production by antigen-specific CD4^+^ T cells and by monocytes Both CD24^hi^CD27^+^ B cell and CD24^low^CD27^−^ B cell inhibit TNF-*α* production by CD4^+^ T cell equally (IL-10 independent pathway)	[15]

CD22 positive selection	CpG (0.5 *μ*M) + BAFF (20 *μ*M) + rHIL-4 (50 U/mL) for 24 hours	IL-10^+^IL-6^+^ Increased expression of TLR9	—	[61]

CD19 negative selection	Anti-IgA+IgG+IgM (6.5 *μ*g/mL) + IL-21 (50 ng/mL) for 16 hours	CD38^+^CD1d^+^IgM^+^CD147^+^IL-10^+^CD25^+^ IDO^+^	Suppression of autologous CD4^+^ T cell proliferation (GzmB mediated)	[36]

CD19 negative selection	CpG (1 *µ*M) + PPD for 48 hours	CD73^−^CD25^+^CD71^+^IL-10^+^ Enriched in: CD5^+^IgM^+^IgD^−^ IL-10^+^CD27^+^ have less CD27 MFI than IL-10^−^CD27^+^	Suppression of autologous PPD-stimulated CD4^+^ T cell proliferation (IL-10 mediated)	[14]

CD19 negative selection	Ultra-CD40L (5% solution) and 200 IU/mL of IL-4 for 5 days	Increased expression levels of CD39 Downregulated CD73	Suppression of autologous CD4^+^ and CD8^+^ T cell proliferation (likely to be partially mediated by ADO and 5′-AMP production by B cell)	[62]

CD19 positive selection	IL-2 (10 ng/mL) + IL-6 (10 ng/mL) + CpG 2006 (1 *µ*g/mL) + IFN-*α* or IFN-*β* (1,000 U/mL) for 4 days	CD27^int^CD38^+^ plasmablasts from naïve and immature B cells	—	[23]

PBMC or CD43 negative selection	LPS (10 *µ*g/mL) + IL-4 (10 ng/mL) + rHIL-35 single-chain fusion protein (100 ng/mL) for 4 days	IL-10^+^	—	[35]

CD19 positive selection	Irradiated CDw32L expressing anti-CD40 mAb	CD11b^−^CD5^−^CD27^−^IgD^+^CD1d^hi^IL-10^+^	Suppression of autologous T cell proliferation and IFN-*γ* production	[60]

CD19 negative selection	CpG (3 *μ*g/mL) + anti-IgA + IgG + IgM (10 *μ*g/mL) for 48 h	CD11c^+^IL-10^+^ Enriched in CD38^+^CD27^−^ GCs Depleted in CD38^+^CD27^+^ PCs	—	[16]

CD19 negative selection	CpG (10 *μ*g/mL) + rHCD40L for 48 hours	CD5^+^CD27^+^CD138^+^CD38^+^IgD^−^GzmB^+^	Inhibition of proliferation of autologous CD4^+^CD25^−^ T cell (GzmB mediated)	[12]

CD19 negative selection	CpG (0.25 *μ*M) for 4 days	—	Effects on CD4^+^ autologous T cell: Suppression of proliferation and differentiation into Th1 cell Reduction of TNF-*α* and IFN-*γ* production Induction of Foxp3^+^CD4^+^ T cell, Tr1 cell, and Th3 cell Upregulation of CTLA-4 (IL-10, TGF-*β*, IDO, and CTLA-4 mediated)	[37]

CD43 negative selection from tonsils	Adipose tissue derived MSC (ratio B cell : MSC 5 : 1) + anti-IgM (10 *μ*g/mL) + IL-2 (10^3^ IU) + anti-CD40 agonistic mAb (5 *μ*g/mL) for 10 days	CD19^+^CD27^−^CD38^hi^CD24^hi^IL10^+^	—	[63]

CD19 positive selection	CD40L (2 mg/mL) + IL-4 (200 IU/mL) for 4 days	CD24^hi^CD38^hi^CD25^+^CD39^hi^CD73^hi^	Decrease in CD69 expression and proliferation Increase of IL-6 secretion from autologous T effector CD4^+^(ADO-A2AR mechanism)	[64]

CD19 positive selection from cord blood	Irradiated CD40L transfected L cells + CpG and anti-BCR for 4 days	IL10^+^ found either in CD24^hi^CD38^hi^ or CD24^int^CD38^int^	Suppression of proliferation of allogeneic CD4^+^ T cell Suppression of secretion of IFN-*γ*, TNF-*α*, and IL-2 by allogeneic CD4^+^ T cell	[65]

PBMC	50% conditioned media with APRIL for 3 days + CpG (10 *μ*g/mL) for additional 24 hours	IL-10^+^	Decrease of IFN-*γ* and TNF-*α* production from autologous CD4^+^ T cell	[66]

PBMC: peripheral blood mononuclear cells; rH: recombinant human; PPD: purified protein derivative; ADO: adenosine; CTLA-4: Cytotoxic T-Lymphocyte Antigen 4; A2AR: A2A receptor. ^*∗*^Unless otherwise indicated, B cells were isolated from PBMC.
